# Re-examining the Disc-Centric Interpretation of Chronic Low Back Pain: A Narrative Review

**DOI:** 10.7759/cureus.109412

**Published:** 2026-05-22

**Authors:** Ammar Sawaf

**Affiliations:** 1 Acupuncture, University of Salford, Salford, GBR

**Keywords:** biomechanical evidence, chronic pain, fatty infiltration, low back pain, multifidus muscle, paraspinal muscles

## Abstract

Chronic low back pain (CLBP) is among the leading causes of global disability. Magnetic resonance imaging (MRI) of the lumbar spine commonly demonstrates degenerated intervertebral discs and/or disc bulging that is regarded as the key contributor to the development of pain. However, degenerative changes of the disc are prevalent in asymptomatic people, thereby calling into question the specificity of these findings. In addition, the assessment and management of CLBP are currently based predominantly on the concept of the disc as the main source of pain and associated disability. The current narrative review aims to question an exclusive focus on disc pathology and to explore the potential contribution of paraspinal muscle dysfunction and biomechanical factors in CLBP. An electronic literature search of PubMed, Scopus, and Web of Science databases was performed for the relevant English-language articles published between January 1990 and February 2025. Studies were chosen according to established eligibility criteria, which encompassed relevance to CLBP in adults, emphasis on paraspinal muscle morphology, spinal biomechanics, or lumbar disc imaging, and the provision of clinically or biomechanically interpretable results. A narrative synthesis of results was conducted, while no quantitative analysis was performed. MRI of asymptomatic patients commonly shows degenerated intervertebral discs and disc bulging, with no consistent relationship between the presence of degenerative changes and pain intensity. Conversely, changes in the muscles, especially lumbar multifidus muscle atrophy, fatty infiltration, and asymmetry, seem to consistently correlate with the presence of CLBP and functional impairment. The longitudinal data suggest the persistence of muscle dysfunction after the remission of pain episodes in CLBP patients. Further, studies on the biomechanics of the spine showed the impact of muscle dysfunction on spinal load distribution and intradiscal pressure (the internal pressure within the intervertebral disc that reflects load transmission through the spine), suggesting that disc bulging may, in some cases, represent a biomechanical adaptation. Degenerative findings are non-specific and should be interpreted considering functional and biomechanical factors. Greater consideration of muscular and biomechanical factors may complement imaging findings in the assessment and management of CLBP. Given the narrative design and predominance of observational evidence, the findings of this review should be interpreted as hypothesis-generating and should not be considered definitive evidence of causal relationships.

## Introduction and background

Chronic low back pain (CLBP) is a widespread health issue, as well as the most common cause of disability worldwide [1,2]. Prevalence rates of CLBP are remarkably high in developed countries, with approximately two-thirds of the adult population being diagnosed with low back pain during their lifetimes [3,4]. Besides the direct cost of CLBP care, lost productivity significantly increases indirect expenses. While CLBP was mostly prevalent in patients aged 35-55 years old in the past, recent studies suggest a possible increase in the number of young adults reporting CLBP, which has been associated with factors such as reduced physical activity, work-related stress, and poor physical fitness, although evidence remains heterogeneous [1,5,6]. Magnetic resonance imaging (MRI) and computed tomography (CT) scanning, to a smaller degree, are now commonly used in the diagnosis of patients with CLBP [1,7]. Intervertebral disc degeneration, disc protrusion, and facet joint arthropathy are common imaging findings in CLBP patients and are considered the main sources of pain [1]. Nevertheless, the association between radiological abnormalities and CLBP has recently become a subject of controversy [8]. In specific contexts, such as radiculopathy, nerve root compression, annular tears, Modic changes, and inflammatory or chemically mediated discogenic pain, intervertebral disc pathology remains a well-established source of symptoms. Degenerative disc changes include structural alterations (e.g., height loss, bulging, signal changes), while paraspinal muscle dysfunction involves changes in muscle size, composition, and neuromuscular control.

Autopsies and imaging tests using myelography, CT, and MRI have consistently demonstrated that structural spinal changes are frequently observed even in individuals without symptoms of low back pain [8]. Extensive population studies and meta-analyses consistently demonstrate that degenerative spinal changes are highly prevalent in asymptomatic individuals and increase with age [3,9]. Degenerative discs, decreased disc signal, decreased disc height, protruding discs, and facet joint arthritis have been observed in up to 90% of asymptomatic subjects aged 60 years or above [3]. Importantly, in specific clinical contexts, including radiculopathy, nerve root compression, annular tears, or advanced degenerative disease, disc pathology remains a well-established and clinically significant source of symptoms. Greater than half of asymptomatic individuals aged 30-39 years already demonstrate disc degeneration or protrusion [3], suggesting that these observations may not represent disease-specific features when considered in isolation but rather age-related or adaptive processes. Evidence regarding the association between disc morphology and pain intensity is inconsistent and often weak. A meta-analysis examining the relationship between disc herniation dimensions and pain severity revealed inconsistent findings, with no significant association noted across studies [10]. Such findings cast further doubt on the assumption that disc pathology alone can account for CLBP, highlighting the diagnostic limitation of disc-oriented imaging.

Despite the inconsistencies between imaging results and clinical picture, degenerative changes of the spine often result in treatment approaches that do not always provide relief [11]. Post-operative pain in patients with spinal diseases is relatively frequent and poorly understood [12]. In comparison, there are numerous observations concerning the spontaneous resolution of disc herniation of the lumbar spine, whereby conservative treatment is associated with partial or complete resolution in a significant number of cases [13]. It is also noteworthy that clinical improvement occurs before radiological changes and indicates an independent course from disc abnormalities. Observational research on the consequences of MRI use for the early detection of lumbar spine pathology showed that early detection of pathology increases the likelihood of spine surgery, opioid prescriptions, healthcare costs, and lack of clinical improvement [14]. Thus, the current clinical guidelines advise against spinal imaging at the early stages of the disease without the presence of red flags [15]. However, despite the above facts, the disc-oriented interpretation still prevails. In addition to structural and biomechanical factors, CLBP is widely recognised as a multifactorial condition influenced by psychosocial and central mechanisms. These include central sensitisation [16], pain-related fear and avoidance behaviours [17], and broader psychosocial determinants [18,19]. These dimensions interact with biological mechanisms and contribute to pain perception and chronicity [20].

In parallel with the growing recognition of the limitations of disc-centric interpretations, increasing attention has been directed toward spinal biomechanics and neuromuscular control as alternative or complementary mechanisms underlying CLBP. The paraspinal muscles, particularly the multifidus muscle of the lumbar spine, play an important role in governing intersegmental spinal movement and stabilisation within the neutral zone (the range of intervertebral motion around the neutral posture where minimal passive resistance is offered) [21]. Studies using imaging and electromyography have shown multifidus muscle atrophy, a decrease in cross-sectional area (CSA), fatty infiltration, and side-to-side disparity in people suffering from CLBP when compared to their healthy counterparts [22-26]. It is worth noting that multifidus muscle dysfunction has been found to persist even after the resolution of acute pain, possibly as a result of inhibition by pain and changes in motor control strategies [27,28].

Mechanistically, dysfunction of the paraspinal muscles may alter the distribution of mechanical loads across lumbar spinal segments. Impaired muscle coordination or asymmetry can increase compressive and shear forces locally, thereby affecting how loads are shared between active (muscular) and passive (disc and ligamentous) structures. Greater muscular imbalance or poor segmental control can increase local compressive and shear stresses, increase unilateral disc pressure, and reduce load sharing between dynamic and static structures of the spine [29-31]. Due to the fast adaptation of skeletal muscle to neuromuscular changes compared to slow disc adaptation to mechanical forces, muscle dysfunction is biologically plausible as a contributing factor to altered spinal biomechanics. In such cases, the occurrence of disc bulging can be seen as a consequence of mechanical adaptation to chronic imbalanced loading rather than a primary nociceptive injury.

Given these considerations, a broader conceptual framework that integrates structural, muscular, and biomechanical perspectives may be required to better understand CLBP. This narrative review reevaluates the predominantly disc-centric interpretation of CLBP by synthesising evidence from imaging, biomechanics, and clinical studies, with particular emphasis on the role of paraspinal muscle dysfunction and its potential contribution to spinal loading alterations and disc changes. For clarity, the review is structured into three main thematic sections, namely, (1) the paradox of spinal degeneration in asymptomatic individuals, (2) paraspinal muscle alterations in CLBP, and (3) biomechanical mechanisms linking muscular dysfunction to secondary disc changes, followed by a critical discussion of their clinical implications. This review adopts an integrative perspective, positioning disc pathology, muscular dysfunction, and psychosocial influences as interacting contributors rather than mutually exclusive explanations. Recent international frameworks and clinical guidelines further emphasise a multidimensional approach to CLBP, integrating structural, functional, and psychosocial factors within a biopsychosocial model of care.

## Review

Methodology

Given the conceptual and integrative aims of this study, a narrative review approach was adopted. This design synthesises interdisciplinary evidence and theoretical perspectives that strictly systematic methodologies may not capture. However, this approach prioritises conceptual interpretation over exhaustive study inclusion.

Search Strategy

The literature search was performed using Scopus, PubMed, and Web of Science electronic databases to uncover peer-reviewed articles written in the English language published between January 1990 and February 2025. The search strategy combined Medical Subject Headings (MeSH) and free-text terms using Boolean operators. A typical PubMed search string was structured as follows: ("chronic low back pain" OR "CLBP") AND ("paraspinal muscles" OR "multifidus" OR "muscle atrophy" OR "fatty infiltration") AND ("spinal biomechanics" OR "intradiscal pressure" OR "disc degeneration"). Equivalent adaptations were applied to Scopus and Web of Science. Detailed search terms used were explained in the Appendices.

Eligibility Criteria

Studies were eligible for inclusion if they (1) involved adult populations (≥18 years) with CLBP or asymptomatic controls; (2) assessed paraspinal muscle morphology, spinal biomechanics, or lumbar disc imaging characteristics; and (3) reported outcomes with direct clinical or biomechanical relevance, defined as measurable associations with pain, disability, functional impairment, or spinal load distribution. Eligible study designs included observational (cross-sectional, cohort, case-control), imaging-based, and biomechanical modelling studies. Studies focusing exclusively on acute injury, infection, malignancy, inflammatory disorders, paediatric populations, or post-operative outcomes without biomechanical relevance were excluded. No formal quality assessment or risk-of-bias evaluation of the included studies was conducted, consistent with the narrative review methodology.

Study Selection Process

Study selection was conducted through iterative screening of titles, abstracts, and full texts based on predefined eligibility criteria. Relevance was operationalised based on alignment with the review objectives, including investigation of muscle morphology, biomechanics, or disc imaging in CLBP. Although formal dual screening was not performed, widely cited and methodologically robust studies were prioritised. This approach may introduce selection bias; however, efforts were made to enhance transparency and conceptual consistency.

Data Extraction

Data extraction was conducted using a structured framework developed for this review. Extracted variables included study design, population characteristics, imaging or biomechanical methods, muscle morphology measures (e.g., CSA, fatty infiltration), spinal loading parameters, and reported associations with pain or functional outcomes. Information was systematically recorded to ensure consistency across the included studies.

Narrative Synthesis

A thematic narrative synthesis was conducted, grouping studies into three domains: (1) spinal degeneration in asymptomatic populations, (2) paraspinal muscle alterations, and (3) biomechanical links between muscle dysfunction and disc changes. Within each domain, findings were compared based on consistency, methodology, and strength of association, with greater weight given to longitudinal studies and those providing converging biomechanical and clinical evidence. Consistent with the narrative review methodology, synthesis emphasised interpretive integration rather than reproducibility or statistical pooling [32]. No meta-analysis was performed. Variability in study design, populations, imaging modalities, and outcome definitions was considered when interpreting overall patterns.

Results

Table 1 illustrates the selected studies that form each theme as representative examples rather than an exhaustive list of all included literature. Where quantitative findings are reported, these reflect results as presented in the original studies and are interpreted descriptively rather than as pooled or directly comparable estimates.

**Table 1:** Representative studies supporting the major themes identified in this narrative synthesis MRI: magnetic resonance imaging; LBP: low back pain; CLBP: chronic low back pain

Core thematic domain	Representative studies	Study design	Population	Key findings relevant to CLBP
Paradox of spinal degeneration versus asymptomatic findings	Jensen et al., 1994 [8]	MRI observational study	Asymptomatic volunteers	Disc bulges and protrusions are common without back pain
	Boden et al., 1990 [9]	Prospective MRI study	Asymptomatic adults	Lumbar disc abnormalities are frequent in pain‑free individuals
Selective paraspinal muscle atrophy and fatty infiltration as functional markers of CLBP	Hides et al., 1995 [24]	Longitudinal MRI study	Acute and chronic LBP patients	Selective multifidus atrophy localised to symptomatic segments
	Kjaer et al., 2007 [25]	Cross‑sectional MRI study	General population	Paraspinal fat infiltration associated with LBP
	Hides et al., 1996 [27]	Longitudinal follow‑up study	LBP patients	Multifidus dysfunction persists despite pain resolution
Biomechanical mechanisms linking muscle dysfunction to secondary disc changes	Panjabi, 1992 [33]	Biomechanical model	Not applicable	Neutral zone expansion links muscle dysfunction to instability
	Wilke et al., 1999 [30]	In vivo experimental study	Healthy volunteers	Muscle activation strongly influences intradiscal pressure
	Dreischarf et al., 2015 [31]	Computational modelling	Simulated lumbar spine	Asymmetric muscle loading increases localised disc stress

The Paradox of Spinal Degeneration Versus Asymptomatic Findings

Evidence shows a paradox in which, despite the high incidence of the appearance of degenerative changes in the imaging tests, patients often remain asymptomatic. This problem was already noted during early research, where researchers pointed to the high incidence of the development of intervertebral discs in pain-free individuals. In a study involving 33 individuals without a history of back pain, a total of 39% had posterior disc protrusions [34]. In another study, researchers found lumbar intervertebral disc abnormalities in 24% of 300 asymptomatic individuals subjected to myelograms [35]. Further CT scan analysis indicated that disc herniation was present in 19.5% of asymptomatic patients younger than 40 years and in 26.9% older than 40 years [36]. Subsequent MRI-based investigations confirmed a high prevalence of lumbar spine abnormalities in individuals without back pain [8].

The above findings have been confirmed by more recent studies, which indicated that asymptomatic degenerative changes in the spine are common, with limited predictive values for CLBP [37]. Particularly, a prospective investigation, conducted among asymptomatic subjects with disc abnormalities in the lumbar spine by MRI scans, indicated that workplace physical and psychosocial factors were important determinants of low back pain-related consultations as well as work incapacity, rather than the imaging characteristics [38]. The findings are corroborated by a large-scale systematic review on the prevalence of MRI and CT findings among asymptomatic subjects [3]. Specifically, in the aforementioned study, the prevalence of disc degeneration had increased significantly with age, from 37% at age 20 to 96% at age 80 [3]. Likewise, the prevalence of the following conditions was shown to increase with age: disc signal change, disc height loss, disc bulge, and facet arthropathy [3].

Another aspect of this paradox is that there is a weak and inconsistent correlation between disc anatomy and pain severity. According to a systematic review involving 31 studies and 2,400 patients, there was very high variability of correlations between the size of herniated discs and pain [10]. Although some studies revealed a positive correlation, other researchers did not find any relationship, inverse relationship, or even inconsistent results [10]. This finding is to imply that the relationship between the size of a herniated disc and pain severity is inconclusive; thus, the role of disc-centric imaging is limited in the absence of neurological defects [10]. Given the limitations of structural imaging in explaining pain, attention has increasingly shifted toward functional and neuromuscular contributors.

Paraspinal Muscle Atrophy and Fatty Infiltration in CLBP

Imaging and observational studies have reported alterations in paraspinal muscle structure in individuals with CLBP, particularly involving the lumbar multifidus muscles. Reported changes include reduced CSA, increased fatty infiltration, and asymmetry compared with asymptomatic populations [21-23]. A consistent finding across imaging studies is that degenerative spinal changes are highly prevalent in asymptomatic populations and demonstrate weak or inconsistent associations with pain severity. However, variability in imaging techniques, measurement protocols, and definitions of muscle quality across studies should be considered when interpreting these findings.

In one study that included 50 patients suffering from CLBP and 40 asymptomatic individuals, multifidus CSA and symmetry were analysed for each lumbar vertebral level [22]. It was found that patients with CLBP had a significantly lower multifidus CSA in the two lowest lumbar vertebrae than asymptomatic participants [22]. This claim is confirmed by other scientific literature, where higher-level research was conducted. Thus, one systematic review including 21 papers concluded that patients with CLBP had a significantly lower CSA and fatty infiltration of the multifidus muscle compared to control groups [23]. Of all paraspinal muscles, the multifidus muscle changes are suggested to be the most consistent and repeatable [23]. Based on that, multifidus muscle morphology was considered a potential diagnostic marker and therapeutic approach for CLBP [23].

A consistent finding across imaging studies is that degenerative spinal changes are highly prevalent in asymptomatic populations and demonstrate weak or inconsistent associations with pain severity. The MRI study conducted in a community population showed a significant association between fatty infiltration of paraspinal muscles and CLBP, physical disabilities, and accompanying structural abnormalities in the spine [38]. An even more sophisticated imaging approach using water-fat MRI revealed that both the extent and spatial pattern of fatty infiltration in the paraspinal muscles were able to distinguish patients with axial CLBP from the asymptomatic subjects, implying the possible application of this feature as an imaging biomarker for CLBP diagnosis [39]. These findings indicate that selective atrophy and fatty infiltration of the paraspinal muscles are commonly associated with CLBP across multiple studies, although the strength and direction of these associations vary depending on study design and methodology. These observed muscular alterations have prompted further investigation into their potential biomechanical consequences.

Muscle-Driven Biomechanical Pathways to Secondary Disc Changes

Biomechanical and experimental studies have demonstrated associations between paraspinal muscle function and spinal loading patterns. These studies report that muscle activity influences intradiscal pressure and contributes to spinal stability [30,33]. Altered muscle activation patterns have been associated with changes in compressive and shear forces acting on lumbar motion segments [30]. Computational models further indicate that asymmetrical muscle loading may result in uneven stress distribution across intervertebral discs [26]. The spine has been modelled theoretically as a system that consists of an active muscular system, a passive disc and ligaments, and a neurodynamic control system. Any dysfunction of the active muscular system, such as impaired function of the lumbar multifidus muscle, is associated with increased neutral zone size, making passive structures responsible for spinal stability [29,33]. However, many of these findings are derived from experimental, computational, or cadaveric models, and direct translation to clinical pain mechanisms should be interpreted with caution.

Biomechanical modelling also lends support to these ideas, showing that an asymmetrical pattern of muscle contraction within paraspinal muscles is associated with asymmetrical loading and increased stress on the discs [31]. Since skeletal muscles adapt to neuromuscular changes faster than the intervertebral discs due to mechanical loads, these observations are consistent with the hypothesis that muscle dysfunction may contribute to altered spinal loading patterns, although causal direction cannot be definitively established based on the available evidence. The hypothesis receives confirmation both from the high incidence of spontaneous regression of lumbar disc herniation during conservative treatment [13] and from the common occurrence of separation between the morphological appearance of a disc and patient complaints [3,8]. Thus, in some cases, disc bulging may represent a response to altered spinal loading patterns; however, the direction of this relationship remains difficult to determine based on current evidence. While these biomechanical mechanisms provide a plausible explanatory framework, detailed modelling assumptions and experimental conditions may not fully reflect clinical complexity.

Discussion

This narrative review re-examined current evidence on the relationships between spinal degeneration, paraspinal muscle alterations, and biomechanical factors in CLBP, with the following section providing an integrated interpretation of these findings. In all these areas, there is sufficient evidence demonstrating that although the presence of degenerative processes in the spine images is frequent, they do not represent a specific factor in relation to pain; on the other hand, the paraspinal muscle structure alterations and their function demonstrate relatively consistent associations with the chronic condition. Interpretation of the findings in this review must be made cautiously. The majority of the included studies are observational, cross‑sectional, or biomechanical in nature, which limits causal inference. While paraspinal muscle alterations demonstrate consistent associations with CLBP and are supported by biomechanical plausibility, this review does not establish direct causality between muscle dysfunction, disc changes, or pain. Taken together, the current evidence base supports a multifactorial interpretation of CLBP, in which disc pathology, muscular dysfunction, biomechanical alterations, and central and psychosocial mechanisms interact rather than operate in isolation. Hence, the evidence should be interpreted as complementary rather than support for a single dominant explanatory model.

Population-based evidence consistently demonstrates that degenerative spinal findings are highly prevalent in asymptomatic individuals and increase with age [3,8,9]. These data challenge the notion that degenerative disc conditions represent pathological processes. Furthermore, reviews addressing the correlation between morphological abnormalities of spinal discs and the level of pain intensity found no consistent associations between disc morphology and pain severity [10], thus questioning the significance of disc-centric imaging when there are no progressive neurological symptoms. These data support the findings of a greater impact of psychosocial and work-related risk factors on pain development compared with the initial imaging findings [14] and current guidelines against routine imaging for uncomplicated low back pain [15]. Unlike degenerative changes in spinal discs, paraspinal muscle changes can be considered reliable indicators of CLBP. Several studies have shown a reduction in multifidus CSA, increased fat content, and muscle morphology asymmetry in patients with chronic pain [22,23]. Among all paraspinal muscles, the multifidus demonstrates the highest sensitivity and specificity in revealing morphological abnormalities, with segmental location usually matching the site of pain localisation [23]. Muscular changes are associated not only with pain but also with disability and functional impairment [21,38].

Additional longitudinal research continues to provide support for the muscle-centric theory. Pain-induced inhibition of the multifidus is quickly initiated in the presence of nociception and fails to revert even when symptoms resolve [21]. The maintenance of this neuromuscular dysfunction serves as a likely cause for the recurrence seen in CLBP and implies that muscle dysfunction could play a role in contributing to chronic back pain, rather than being an adaptive reaction to the condition. Research showing symptom improvement through targeted muscular training without any alteration in disc structure supports this theory [27].

Research from biomechanics is able to explain a mechanical relationship between paraspinal muscle dysfunction and secondary effects on discs. Experimental data revealed the significant effect of muscle activity on intradiscal pressure and loading in the vertebral column [30]. There is an approach that treats the spine as a multi-subsystem mechanism and includes active, passive, and neurodynamic components, where muscular dysfunction may contribute to an enlarged neutral zone, higher segmental mobility, and increased load on the passive component of the spine [29-33]. Computer modelling also confirmed that asymmetrical or reduced activity of the muscles has been linked to load imbalance, leading to focal disc stress [31]. Considering the rapid adaptation of muscle tissue in comparison with intervertebral discs, it is plausible that muscle dysfunction may precede or contribute to the development of disc deformations. These findings should be interpreted within a broader clinical and conceptual framework that considers multiple interacting mechanisms.

The observations from routine rehabilitation practice, presented for contextual illustration rather than empirical validation based on rehabilitation, are also aligned with this interpretation and existing scientific literature. The patients have been demonstrating inconsistency between the results of the imaging studies and the severity of their symptoms, as well as a substantial clinical improvement after applying treatments aimed at increasing muscle activation, balance, and resistance in the paraspinal region without any observable changes on the imaging [28,40,41]. These clinical data are provided here to provide background for, not to substitute, the imaging and biomechanical evidence analysed within this review. Overall, the evidence synthesised within this review highlights the limitations of disc‑centric interpretations when used in isolation. These observations are not intended to substitute controlled evidence but are discussed to contextualise the imaging and biomechanical findings reviewed. The conceptual differences between these perspectives are described in Table 2.

**Table 2:** Contrasting emphases in disc-focused and muscle-focused interpretations of chronic low back pain Note: These perspectives are presented as complementary rather than mutually exclusive. MRI: magnetic resonance imaging

Feature	Disc-centric model	Muscle-centric model
Primary pathology	Disc degeneration or bulging [3,8,9]	Paraspinal muscle dysfunction [21-23]
Diagnostic focus	Static MRI findings [3,8,10]	Functional and morphological muscle assessment [21-23,38]
Symptom correlation	Weak and inconsistent [3,8,10]	Stronger and more reproducible [22,23,38]
Time-related relationship	Structural disc changes assumed to precede pain [10,37]	Muscle dysfunction may precede disc changes [21,27,31]
Explanation for asymptomatic degeneration	Limited explanatory power [3,8,9]	Interpreted as a biomechanical adaptation [21,31,33]
Therapeutic emphasis	Surgery and symptom-focused pharmacological treatment [11,14]	Neuromuscular and motor control-based rehabilitation [21,23,27]

The present model further proposes that persistent paraspinal muscle stiffness may represent a primary and under-recognised contributor to non-specific CLBP. Sustained muscle shortening and increased tone may mechanically approximate adjacent vertebral segments, altering load distribution and increasing localised intradiscal pressure, which may contribute to the development of secondary disc bulging. In parallel, prolonged muscle contraction may impair local microcirculation, creating a relative ischemic environment with the accumulation of metabolic by-products such as lactate and protons, which are known to sensitise nociceptive pathways. Within this framework, muscle stiffness is not only a mechanical factor but also a potential biochemical driver of pain generation in CLBP.

These findings will clearly have important implications not only clinically but also for future research. In terms of the former, an excessive focus on imaging of the spine alone will result in an undue emphasis on the degenerative condition of the spine as the primary cause of symptoms. The inclusion of parameters relating to muscle quality, neuromuscular control, endurance, and muscle asymmetry will help narrow down the diagnosis and potentially help determine whether or not there are any modifiable factors contributing to the problem. Clinically speaking, this would suggest a need for more emphasis on conservative therapy focused on muscles in terms of motor control as well as stabilising exercise training and less need for early invasive surgery in cases where no neurological deterioration is seen in the patient. This approach can be seen as in agreement with currently existing guidelines focusing on the conservative and functional treatment of CLBP. In terms of research, future efforts should focus on longitudinal studies involving imaging and biomechanics of the musculature, which might enable more personalised treatment. These considerations should be interpreted in alignment with existing clinical guidelines and individual patient presentation.

The present narrative review has some limitations that should be acknowledged. As a narrative review, the selection and synthesis of literature may be subject to interpretive and citation bias, and efforts were made to include representative studies across differing explanatory frameworks. The majority of the included studies are observational or biomechanical in nature, limiting causal inference. The possibility that paraspinal muscle changes represent secondary adaptations to pain, disuse, or behavioural factors cannot be excluded. In addition, psychosocial, cognitive, and central sensitisation mechanisms were not addressed, despite their known contribution to CLBP. Finally, heterogeneity in imaging techniques, outcome measures, and study populations may influence the apparent consistency of findings across studies.

## Conclusions

Degenerative findings of the lumbar spine are commonly observed on routine imaging in both patients with CLBP and asymptomatic individuals, and they often represent incidental or adaptive changes rather than the primary source of pain, thereby limiting the diagnostic value of structural abnormalities. In contrast, paraspinal muscle dysfunction, particularly of the multifidus, shows a more consistent association with pain and functional impairment, with disc bulging viewed as a secondary biomechanical consequence and therapeutic management guided by functional assessment and neuromuscular rehabilitation. These findings support further investigation into muscle‑focused assessment and rehabilitation strategies as complementary components of CLBP management, rather than as replacements for existing diagnostic or therapeutic paradigms. Importantly, CLBP remains a multifactorial condition, with psychosocial, neurological, and behavioural factors also playing significant roles.

## Appendices

**Table 3:** Search strategies and search terms WoS: Web of Science

Database	Search date	Search terms
PubMed	01 Jan 1990 to 31 Dec 2025	("chronic low back pain"[MeSH Terms] OR "chronic low back pain"[Title/Abstract] OR CLBP[Title/Abstract]) AND ("intervertebral disc degeneration"[MeSH Terms] OR "disc degeneration"[Title/Abstract] OR "discogenic pain"[Title/Abstract]) AND ("paraspinal muscles"[MeSH Terms] OR "lumbar multifidus"[Title/Abstract] OR "erector spinae"[Title/Abstract]) AND ("muscle weakness"[Title/Abstract] OR "fatty infiltration"[Title/Abstract] OR "muscle atrophy"[Title/Abstract] OR "muscle imbalance"[Title/Abstract]) AND ("biomechanics"[MeSH Terms] OR biomechanics[Title/Abstract] OR "spinal stability"[Title/Abstract])
Scopus	01 Jan 1990 to 31 Dec 2025	(TITLE-ABS-KEY("chronic low back pain" OR CLBP)) AND (TITLE-ABS-KEY("intervertebral disc degeneration" OR "disc degeneration" OR "discogenic pain")) AND (TITLE-ABS-KEY("paraspinal muscle*" OR "lumbar multifidus" OR "erector spinae")) AND (TITLE-ABS-KEY("muscle weakness" OR "fatty infiltration" OR atrophy OR imbalance)) AND (TITLE-ABS-KEY(biomechanic* OR "spinal stability"))
WoS	01 Jan 1990 to 31 Dec 2025	TS=("chronic low back pain" OR CLBP) AND TS=("intervertebral disc degeneration" OR "disc degeneration" OR "discogenic pain") AND TS=("paraspinal muscle*" OR "lumbar multifidus" OR "erector spinae") AND TS=("muscle weakness" OR "fatty infiltration" OR "muscle atrophy" OR imbalance) AND TS=(biomechanic* OR "spinal stability")
